# Supplementary material for: Empirical methods for controlling false positives and estimating confidence in ChIP-Seq peaks
Source: BMC Bioinformatics. 2008 Dec 5;9:523. doi: 10.1186/1471-2105-9-523 (PMC2628906; doi:10.1186/1471-2105-9-523)
Supplement: Additional File 1 — A variety of html documents from the USeq web site detailing the available applications, their best usage, output file type descriptions, command line menus, etc. [file 1471-2105-9-523-S1.zip › USeqUserGuides/JavaDoc.zip!/JavaDoc/util/apps/package-summary.html]

util.apps


|  |  |  |
| --- | --- | --- |
| **Overview** \| **Package** \| Class \| **Use** \| **Tree** \| **Deprecated** \| **Index** \| **Help** | |  |
| **PREV PACKAGE**   **NEXT PACKAGE** | **FRAMES**    **NO FRAMES**     **All Classes** |  |


---

## Package util.apps

Scripts for printing arrays, manipulating files, scatter plots...

**See:**
  
          **Description**

| **Class Summary** | |
| --- | --- |
| **ArrayPrinter** | Prints serialized arrays to screen. |
| **Correlate** | Calculates a Pearson correlation coefficient between all pairs of serializae float[]. |
| **Delme2** | For testing |
| **FileJoiner** | Joins text files into a single file, avoiding line concatenations. |
| **FileSplitter** | Application for splitting a large text file into smaller files given a maximum number of lines. |
| **FileTrimmer** | Removes empty lines from text files in a given directory. |
| **FilterAgilentProbeFile** | Assumes one chromosome. |
| **Flash** |  |
| **GeneCentricCNVParser** |  |
| **GeneNameCounter** | Counts the frequency of words in the master list, then assigns these frequencies to words in the test list. |
| **HouseBean** |  |
| **IntersectLists** | Compares two lists of words for intersection. |
| **JQSub** | Fires command lines on clusters through qsub. |
| **MakeBioTools** | Utility app to pull classes into folders and extract menus to html. |
| **MergeColumns** |  |
| **ParseMLSHTML** |  |
| **ParserForGff** |  |
| **PrintSelectColumns** | Prints out select columns from a tab delimited file, skips lines, appends words, etc. |
| **RenameRWSResults** | Takes the output of RocWindowScanner from the cluster and renames the file |
| **ScatterDrawFrame** | Helper class for `ScatterPlot`, the frame. |
| **ScatterDrawPanel** | Helper class for `ScatterPlot`, the guts of drawing the scatter plot. |
| **ScatterPlot** | Draws a simple scatter plot and calculates a Pearson correlation coefficient for two serialized int[] or float[] arrays. |
| **ScoreChromosomes** | Scores chromosomes for the presence of transcription factor binding sites. |
| **ScoreSequences** | Scores sequences for the presence of transcription factor binding sites. |
| **SmoothData** | Pulls the first two columns of double from a white space delimited file. |
| **StatBarFiles** |  |
| **SubtractOneFromEachColumn** | Subtracts 1 from each column assumes integers. |
| **TASSignalChromFileSplitter** |  |
| **TextParser** | For parsing a text file into a tab delimited file, working template. |
| **WindowScoreSmoother** | Slides a window across an array of numbers averaging the scores. |
| **ZipAndDelete** |  |

## Package util.apps Description

Scripts for printing arrays, manipulating files, scatter plots...

---


|  |  |  |
| --- | --- | --- |
| **Overview** \| **Package** \| Class \| **Use** \| **Tree** \| **Deprecated** \| **Index** \| **Help** | |  |
| **PREV PACKAGE**   **NEXT PACKAGE** | **FRAMES**    **NO FRAMES**     **All Classes** |  |


---
